# The influenza A (H1N1) pandemic in Reunion Island: knowledge, perceived risk and precautionary behaviour

**DOI:** 10.1186/1471-2334-13-34

**Published:** 2013-01-24

**Authors:** François Taglioni, Michel Cartoux, Koussay Dellagi, Cécile Dalban, Adrian Fianu, Fabrice Carrat, François Favier

**Affiliations:** 1 University of Reunion Island, BP 7151, Saint-Denis cedex 9, Reunion Island, 97715, France; 2 Centre de recherche et de Veille sur les Maladies Emergentes dans l’Océan Indien (CRVOI). Plateforme de Recherche CYROI, 2 Rue maxime Rivière, Sainte-Clotilde Cedex, Reunion Island, 97491, France; 3 IRD, Représentation de la Réunion, CS 41095, Sainte Clotilde Cedex, Reunion Island, 97495, France; 4 CIC-EC, INSERM, BP 350, Saint Pierre Cedex, Reunion Island, 97448, France; 5 INSERM UMR-S 707, Faculté de médecine Saint-Antoine, 27, rue Chaligny, PARIS cedex 12, France; 6 UMR-S 707, UPMC Université Paris 06, Paris, France; 7 U 707, INSERM, Paris, France

**Keywords:** Influenza A (H1N1) pandemic, Knowledge, Perceived risk, Perceived vulnerability, Precautionary behaviour

## Abstract

**Background:**

The effectiveness of preventive measures depends on prevailing attitudes and mindsets within a population. Perceived risk is central to a shift in mindset and behaviour. The present study aims to investigate the perceived severity, vulnerability and precautionary behaviour adopted in response to the influenza A (H1N1) epidemic that broke out in 2009 on Reunion Island (Indian Ocean). As no H1N1 vaccination was available at the time, non-medical interventions appeared of crucial importance to the control of the epidemic.

**Methods:**

A cross sectional survey was conducted in Reunion Island between November 2009 and April 2010 within 2 months of the passage of the influenza A (H1N1) epidemic wave. Individual contacts representing 725 households (one contact per household) were interviewed by telephone using validated questionnaires on perceived risks. Mean scores were calculated for perceived severity, vulnerability, efficacy of preventive measures and precautionary behaviour. Univariate analysis was applied to identify preventive measures and attitudes and multivariate analysis was used to study the determinants of precautionary behaviour.

**Results:**

More than 95% of contacted persons accepted to participate to the survey. Eighty seven percent of respondents believed that prevention was possible. On average, three out of six preventive measures were deemed effective. Spontaneously, 57% of the respondents reported that they took one or more preventive measures. This percentage increased to 87% after the interviewer detailed possible precautions one by one. The main precautions taken were frequent hand washing (59%) and avoidance of crowded places (34%). In multivariate logistic regression analysis the following factors were significantly associated with taking one or more preventive measures: young age, previous vaccination against seasonal influenza, having had seasonal influenza in the last five years, effectiveness of the preventive measures taken and low standards of education.

**Conclusion:**

Inhabitants of Reunion Island have expressed a preventive approach adapted to the realities of the H1N1 pandemic, a feature that likely reflects some preparedness gained after the large and severe chikungunya epidemic that hit the island in 2006. The degree of severity was well assessed despite the initial alarmist messages disseminated by national and international media. Precautions that were undertaken matched the degree of severity of the epidemic and the recommendations issued by health authorities. Further qualitative studies are needed to help adapting public messages to the social and cultural realities of diverse communities and to prevent misconceptions.

## Background

Compliance with preventive measures, e.g. non-medical action, is dependent on the attitude and willingness of the population and on the specific actions recommended by health authorities [1-3]. Precautionary behaviour results from a combination of social and psychological factors such as personal values, socio-economic status and cultural background, gender, education, knowledge, and beliefs about the disease, including perceived risks and perceived effectiveness of the proposed action [2][4-6]. These factors may be specific to each target population and should be investigated to develop a locally adapted approach [7,8]. Understanding perceptions and reactions among the general public during pandemics may improve information and communication about health risks and help shifting attitudes among the general public [9-11].

The outbreak of a new influenza A (H1N1) virus started in Mexico and the United States at the end of April 2009 and quickly spread to other countries. On 11 June 2009 the World Health Organization (WHO) declared a global influenza A (H1N1) pandemic, thereby raising major international concern over the risk of high morbidity and mortality [12]. Some 14,000 deaths related to influenza A (H1N1) were reported worldwide in the period up to January, 2010 [13].

Reunion Island is a subtropical overseas French Island in the southern hemisphere, in the S.W. Indian Ocean, lying 700 km east of Madagascar and 200 km S.W. of Mauritius. The Reunion Island population of 810.000 is composed with communities from various ethnic origins (European, African, Asian) [14]. The first case due to influenza A (H1N1) virus was detected in a traveler returning from Australia on July 5, 2009 [15]. The first autochthonous case was reported on July 21, 2009 and the influenza A (H1N1) epidemic broke out during the normal period of seasonal influenza ie; austral winter. Hence, the outbreak started on week 30 (July 20), peaked on week 35 (August 28) and lasted until week 38 (September 20). A serological survey conducted on Reunion Island estimated the seroconversion rates to the pandemic virus at 45.2% (all ages) and at 63.2%, 39.4%, 16.7% in the <20 years, 20–59 years and ≥60 years old respectively [16]. During the outbreak, 14 death certificates reporting influenza-like illness were reported to the island’s public health authorities. The 2009 pandemic influenza A (H1N1) had no detectable impact on the overall mortality on Reunion Island since no excess of mortality was observed during the outbreak [17].

The context in which the Reunion Island population was challenged by the H1N1 pandemic is worth considering: In 2005–2006, an epidemic of chikungunya, a vector borne infectious disease due to an alpha virus transmitted by the mosquito *Aedes Albopictus*, originated from the East African coast and diffused in all the Indian Ocean region. The epidemic had a major public health impact in Reunion Island (over one third of the population was infected) [18] and received extensive media coverage [19]. As a result of the chikungunya epidemic, Reunion Island’s population has been sensitized and prepared to the dangers associated with emerging epidemic diseases.

At the time the epidemic started in Reunion Island (July 21, 2009) no specific vaccine against the H1N1 virus was available (the vaccine became available in November, 2009). Hence health authorities had turned their attention to non-medical measures recognized as having an impact on infection transmission and mortality rates [20]. A local campaign emphasized regular hand washing and avoidance of contact with diseased persons. They recommended covering the mouth and nose with a paper tissue and wearing a mask if infected. The general public was encouraged to consult a doctor as soon as symptoms of respiratory infection appeared and to stay at home and take individual protective measures [17].

The present study aimed to investigate perceived risks, concern, behavioural responses and other key determinants of precautionary behaviour related to the outbreak of the influenza A (H1N1) in Reunion Island. This was done in the frame of a research programme called CoPanFlu-RUN that included three complementary components of epidemiological, virological and social science aspects. The epidemiological and virological parameters of the epidemy were assessed in a prospective study conducted from July 21 to December 23, 2009 during the outbreak of influenza A (H1N1) [16,21,22]. Shortly after passage of the epidemic wave, the social science aspects were specifically investigated in a cross-sectional study.

## Methods

### Participants and sample

Following the H1N1 outbreak, a cross-sectional telephone survey was conducted between November 9, 2009 and April 12, 2010 on perceived risks and precautionary behavior of Reunion Island inhabitants. The minimum sample size was estimated to 474 adults, assuming that 30% of individuals were taking precautionary behaviour, and considering 1% absolute precision and 5% p-value. The participants who accepted to participate to the cross sectional survey were recruited from the cohort investigated by the CoPanFlu-RUN prospective study [16]. This original cohort was composed of 762 households (2164 inhabitants) (for details on the original cohort see [16,3]). Participants in the described study were told that the survey focused on the outbreak of influenza A (H1N1) and their informed written consent was required.

The questionnaire was administrated on a household basis. One household’s reference person was identified among the three oldest members of each household. If there were a senior couple in the household, the reference person was always the male parent. If there was no couple in the household, the reference person was the oldest non-dependent (male or female) person (source: French national institute for statistics and economical studies).

### Questionnaire

The questionnaire used in this study had already been validated in a previous research protocol [23]. The questionnaire was based on an integrated model designed to explain precautionary behaviour, including constructs drawn from the Protection Motivation Theory (PMT) [24] and Health Belief Model (HBM) [25]. The survey about influenza A (H1N1) outbreaks included a wide range of questions related to levels of knowledge, antecedents (infection and vaccination), perceived risks (including perceived severity and perceived vulnerability), response efficacy, perceived self-efficacy and precautionary behaviour.

Regarding knowledge of clinical signs and modes of transmission, scales of 0 to 6 were compiled based on the number of positive responses, resulting in mean scores of symptom knowledge. Firstly, a score reflecting knowledge of the four major symptoms that are frequently encountered in mild influenza (fever, headaches, aches and pains, running nose) and more specifically in respiratory complications (coughing and dyspnea) was deduced from responses to the question: “What are the symptoms of influenza A (H1N1)?”. Secondly, a score estimating knowledge of airborne (saliva and sneezing) and contact-based (hands or objects) viral transmission modes was deduced from responses to the question: “What are the modes of transmission of influenza A (H1N1)?”. Two other questions suggesting erroneous modes of transmission were asked (eating pork meat; stung by mosquitoes).

Regarding antecedents (infection and vaccination), the questionnaire included a question on whether the respondent was vaccinated against seasonal influenza in the last year and whether he or she had suffered from seasonal influenza within the last five years.

Several questions tried to assess the perceived severity of influenza A (a person’s belief of how serious contracting the illness would be for him/her). Three questions were related to the pathology itself: “do you think influenza A is more severe than seasonal influenza?”; “do you think influenza A is a fatal disease?”; “is there no efficient treatment against influenza A?”. Another question was asked in order to quantify the perceived severity. This question “if you were to get infected with influenza A, how serious a health issue would it be for you?” resulting in a score of perceived severity on a scale of 0 to 10. Moreover, a general question addressed the impact on public health of influenza A (H1N1) among the population (percentage of the population infected by influenza A (H1N1), number of deaths).

The question: “how likely are you to get infected with influenza A (H1N1)?” gave a score of perceived vulnerability of influenza A (H1N1) on a scale of 0 to 10. The perceived vulnerability of influenza A (H1N1) is a person’s perception of the chance that he/she will contract the disease. A similar question to assess the vulnerability of seasonal flu gave a score of perceived vulnerability of seasonal flu on a scale of 0 to 10. Also, a list of twelve potential risks or dangers quoted by the media or present in the environment (GMO, cell phones, cyclones, global warming, chikungunya, air pollution, car accidents, smoking, pesticides in food, diabetes, AIDS, cancer) was proposed by the interviewers and the respondents had to rank for each potential risk their concerns with regard to these risks on a scale of 0 to 10.

Response efficacy (a person’s belief in the effectiveness of the preventive measure) was evaluated by the interviewer with two questions. Firstly a general question with a dichotomous answer (yes or no) was asked “are there effective preventive measures to protect you from influenza A (H1N1)?”. Secondly, a list of six recommended influenza A (H1N1) prevention measures was read (washing hands more frequently, getting vaccinated against seasonal influenza, wearing masks in public, avoiding public transport, avoiding crowed places, not sending children to school). The question was “which preventive measures among this list you think are effective at keeping you from getting the influenza A (H1N1)?”. The effectiveness of each preventive measure had been ascertained separately. Furthermore, precautions regarded as effective by each individual produced a score for the effectiveness of preventive measures from 0 to 6.

Perceived self-efficacy (a person’s level of confidence in his/her ability to perform the preventive measure) was assessed by asking a question with a dichotomous answer (sure or not sure) “how sure are you that you can by yourself prevent getting the influenza A (H1N1)?”.

During the interview, respondents were first asked whether they had taken precautions to avoid influenza during the epidemic. All responses were recorded, including those which did not correspond to official recommendations from health authority or mistaken responses. In a second, more directive phase, six types of precautions (washing hands more frequently, getting vaccinated against seasonal influenza, wearing masks in public, avoiding public transport, avoiding crowed places, not sending children to school) were mentioned one by one by the interviewer. Respondents were asked whether they had taken measures to prevent them getting infected with influenza A (H1N1). Respectively, two scores of preventive measures, taken immediately and after detailed review with interviewer, were implemented on a scale of 0 to 6.

### Statistical analysis

Data entry used EpiData version 3.1 (The Epidata Association, Odense, Denmark). SAS version 9.1 (SAS Inc., Cary, NC, USA) was used for statistical analysis. To study correlates of precautionary behaviour, a new dichotomous variable “precautionary behaviour” was defined and coded 1 (yes), if respondents had taken one or more preventive measures, and coded 0 (no) if respondents had done nothing. Univariate and multivariate logistic regression analyses were performed to identify factors significantly associated with taking one or more preventive measures. For univariate analysis, demographics, knowledge, antecedents (infection and vaccination) perceived severity, perceived vulnerability, response efficacy and perceived self-efficacy were entered as predictor variables.

First a univariate analysis was performed using chi-squared tests or Fisher’s exact tests for categorical variables and Student’s *t*-test for normally distributed continuous data; non-parametric tests (Mann–Whitney) were used if appropriate. Univariate analyses were performed with self-reported characteristics as independent variables and taking precautionary behaviour as the dependent variable. For the odds ratios, 95% confidence intervals (CI) were calculated. Second, for the multivariate regression analyses, all factors with a p-value <0.2 in the univariate analysis were entered in the multivariate model. The potential confounding factors (age) were included in our multivariate model. This model was fitted with a step-to-step backward elimination.

### Ethical considerations

The questionnaire was conducted in accordance with the Declaration of Helsinki and French law for biomedical research (Nu ID RCB AFSSAPS: 2009-A00689-48) and was approved by the relevant Ethics Committee (Comité de Protection des Personnes of Bordeaux 2 University). Persons eligible for participation were asked to give their written, informed consent.

## Results

### Socio-demographic characteristics and influenza like illness

Among the 762 households constituting the original CoPanFlu-RUN sample that were contacted by telephone, individuals representing 725 households accepted to take part in the survey, giving a participation rate of 95%. There was a majority of women (73%) (versus 52% in the general Reunion island population)^a^. Our sample comprised also a majority of elderly people, 48% over 60 years olds (versus 17% in general Reunion island population)^a^. One third of respondents had graduated from high school and/or were in higher education (Table 1).

**Table 1:** Distribution of general characteristics in the study population

		**N**	**%**
**Overall sample**		**725**	**100%**
**Socio-demographic characteristics**			
**Sex**			
	Male	192	27%
	Female	533	73%
**Age**			
	18-29 years	53	7%
	30-44 years	237	33%
	45-59 years	91	12%
	60 + years	344	48%
**Educational level**			
	High school graduate or higher educational level	214	30%
	Not high school graduate	511	70%
**Influenza: infection, vaccination and antecedents**			
**Infected with influenza during the outbreak 2009**			
	No	462	64%
	Yes	263	36%
**Infected with seasonal influenza during the past five years**			
	No	231	32%
	Yes	494	68%
**Vaccinated against seasonal influenza**			
	No	543	75%
	Yes	182	25%

Over one third of the respondents (36%) stated that they had had influenza like illness during the epidemic of 2009 (Table 1). Two thirds (68%) declared that they had had seasonal influenza in the last five years (Table 1). Vaccination against seasonal influenza was reported by 25% of all participants (Table 1), and by 40% of people aged 60 and over.

The youngest respondents (18–59 years) declared having had influenza like illness more frequently than elderly people (43% versus 30%, p < 0.001)^a^. The proportion of individuals declaring influenza like illness declined with age as follows: 53% in 18–29 year olds, 43% (30–44 years), 34% (45–59 years) and 30% (≥60 years)^a^. Fewer elderly people stated they had had influenza like illness in the last five years compared with the younger respondents (p < 0.001). Elderly people were also more frequently vaccinated (p < 0.001)^a^.

### Knowledge of the symptoms and modes of transmission

On the average, knowledge of influenza A (H1N1) symptoms scored 2.35 (SD 1.29) on a scale of 0 to 6 (Table 2). Eighty seven percent of respondents reported at least one correct symptom^a^. The majority of the respondents (78%) felt that they had been well informed about influenza A (H1N1)^a^. Main symptoms identified by respondents were (in decreasing order of frequency): fever (79%), aches and pains (53%), headaches (36%) and a running nose (25%)^a^. Symptoms more specifically related to the respiratory system and more suggestive of severity, (cough and dyspnea) were identified less frequently; 33% and 9.5% respectively^a^.

**Table 2:** Score of knowledge; perceived severity; perceived vulnerability; precautionary behaviour taken

**2.1 Knowledge of symptoms and transmission modes (scale 0–6)**					
		**Taking one or more preventive measures**			
		**(n = 725)**	**(n = 633)**	**(n = 92)**	
		**Overall**	**Yes**	**No**	**p-value**
**Score of symptom knowledge**					
	Mean	2.35	2.35	2.30	0.52
	SD	1.29	1.31	1.19	
	Median	2	2	2	
**Score for knowledge of modes of transmission**					
	Mean	4.47	4.50	4.30	0.324
	SD	1.38	1.36	1.52	
	Median	5	5	4.5	
**2.2 Perceived severity, vulnerability (scale 0–10)**					
		**Taking one or more preventive measures**			
		**(n = 725)**	**(n = 633)**	**(n = 92)**	
		**Overall**	**Yes**	**No**	**p-value**
**Score of perceived severity**					
	Mean	6.05	6.14	5.40	0.017
	SD	3.05	3.03	3.18	
	Median	7	7	7	
**Score of perceived vulnerability**					
	Mean	5.26	5.43	4.10	0.001
	SD	3.67	3.65	3.64	
	Median	7	7	3.5	
**2.3 Effectiveness of preventive measures (scale 0 – 6)**					
		**Taking one or more preventive measures**			
		**(n = 725)**			
		**Overall**			
**Score for the effectiveness of preventive measures**					
Mean		3.04	3.15	2.30	<0.001
SD		1.57	1.55	1.53	
Median		3	3	2	

The score of positive responses to modes of transmission was 4.47 (SD 1.38) on a scale of 0 to 6 (Table 2). Among respondents, 90% and 80% were familiar with airborne transmission (saliva, sputter and cough) and with transmission through contact (hands or objects) respectively^a^. Erroneous modes of transmission were mentioned by 43% of respondents^a^. For example, 9% mentioned pork meat as a potential mode of transmission while 40% referred to the mosquito^a^.

### Perceived severity and vulnerability

Perceived severity scored 6.05 (SD 3.05) whereas perceived vulnerability scored 5.26 (SD 3.67) on a scale of 0 to 10 (Table 2). Perceived severity observed by score was reinforced by the dichotomous question (“do you think influenza A is more severe than seasonal influenza?”). Indeed, a majority (58%) perceived influenza A (H1N1) as more severe than seasonal influenza^a^. Moreover, influenza A (H1N1) was regarded as being untreatable by 46% of the respondents and as fatal by 42%^a^. Respondents felt that 30% of the Reunion Island’s population was infected by influenza A (H1N1)^a^ and that approximately twenty people had died of influenza A (H1N1).

Perceived severity, perceived vulnerability and estimated proportion of individuals infected by influenza differ by sex. Among women, severity score (6.32 versus 5.31, p = 0.001)^a^ and vulnerability scores (5.70 versus 4.72, p < 0.001)^a^ were significantly higher than among men. This trend was also observed with the dichotomous question on perceived severity (60% versus 51%, OR 1.4, p = 0.03) and for the estimated proportion of individuals infected by influenza A (H1N1) (means of 33.20 versus 21.15, p < 0.001)^a^. However, these risk perception indicators did not vary with age.

**Figure 1:**
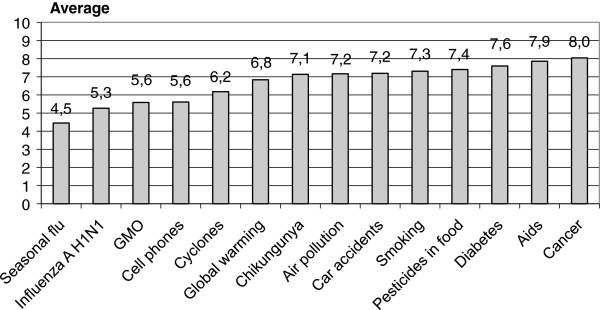
Concerns rated on a scale of 0 to 10 (n=725).

Compared to other concerns, influenza A (H1N1) was not considered as a major cause for concern (level of concern 5.3). In terms of epidemic severity, the chikungunya virus raised more concern than the influenza A (H1N1) (level of concern 7.1). The main health concern issues were, in decreasing order: cancer, AIDS and diabetes (between 7.6 to 8.0 on a scale of 0 to 10) (Figure 1).

### Effectiveness of preventive measures

The effectiveness of preventive measures (response efficacy) scored 3.04 (SD 1.57) on a scale of 0 to 6 and was higher among those who took precautions (3.15 versus 2.30, p < 0.001) (Table 2). This result by score was reinforced by most respondents (82%)^a^ who regarded prevention as possible. Similarly, 83% of respondents felt that it was possible to reduce the risk of infection through individual preventive action (perceived self-efficacy)^a^.

### Precautions taken

**Figure 2:**
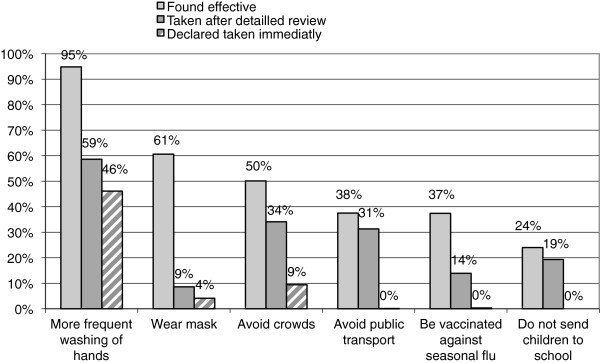
Preventive measures taken and found effective against the H1N1 virus (n=725).

Precautions taken scored 0.60 (SD 0.67) on a scale of 0 to 6 and scored 2.02 (SD 1.19) when the interview reviewed the six available precautions^a^. The majority of the respondents (57%) declared that they had taken at least one precaution against influenza A (H1N1)^a^. This percentage increased to 87% after the interviewer detailed possible precautions one by one^a^. The two main precautions that the respondents stated they took, regardless of the method of questioning, were: more frequent hand washing and avoidance of mixing in groups (Figure 2).

Figure 2 combines for each recognized level of efficacy, the percentage of precautions that the respondents said they had taken (immediately and after listing all the precautions). Hand washing more was the precaution that the respondents considered as the most effective (95%) and appeared to also be the most implemented as a preventive measure against A influenza: 46% spontaneously replied that they had taken this precaution and 59% confirmed they had taken this precaution after it was recalled by the interviewer. Paradoxically, the wearing of masks, although better perceived in terms of efficacy than avoidance behaviour, was not widespread (9%). Vaccination against seasonal influenza was considered as effective (37%) though it was less used (14%) to prevent influenza A (H1N1).

A univariate analysis revealed that eight significant factors contributed to the precautions taken against influenza A (H1N1): 1) being a female (Table 3), 2) having been vaccinated against seasonal influenza (Table 3), 3) having had influenza in the last five years (Table 3), 4) a higher perceived severity (Table 3), a higher score of perceived severity (Table 2), 5) a higher score of perceived vulnerability (Table 2), 6) a higher response efficacy (Table 3), 7) a higher perceived self efficacy (Table 3), 8) a higher score for the effectiveness of preventive measures (Table 2) .

**Table 3:** Proportions of respondents that reported to have taken one or more preventive measures and results from univariate analysis

		**N**	**%**	**OR**	**95% CI**	**p-value**
**Overall sample**		**725**	**87%**			
**3.1 Socio-demographic characteristics**						
**Sex**						
	Male	192	83%	1.00		0.028
	Female	533	89%	1.67	1.02–2.71	
**Age**						
	18–29 years	53	98%	1.00		0.423
	30–44 years	237	85%	1.31	0.81–2.11	
	45–59 years	91	86%	1.70	0.59–2.28	
	60 + years	344	88%	0.97	0.61–1.53	
**Educational level**						
	High school graduate or higher educational level	214	84%	1.00		0.094
	Not high school graduate	511	89%	1.40	0.90–2.30	
**3.2 Influenza: infection, vaccination and antecedents**						
**Infected with influenza during the outbreak 2009**						
	No	462	86%	1.00		0.139
	Yes	263	90%	1.43	0.87–2.30	
**Vaccinated against seasonal influenza**						
	No	543	85%	1.00		<0.001
	Yes	182	96%	3.98	1.88–8.39	
**Infected with seasonal influenza during the past five years**						
	No	231	82%	1.00		0.002
	Yes	494	90%	1.97	1.26–3.07	
**3.3 Perceived severity**						
**Influenza A is more severe than seasonal influenza**						
	No	305	84%	1.00		0.023
	Yes	420	90%	1.65	1.06–2.56	
**Influenza A is a fatal disease**						
	No	423	84%	1.00		0.005
	Yes	302	91%	1.96	1.21–3.17	
**There is no efficient treatment against influenza A**						
	No	395	90%	1.00		0.023
	Yes	330	84%	0.60	0.38–0.96	
**3.4 Response efficacy, perceived self efficacy**						
**Response efficacy**						
	No	131	79%	1.00		0.001
	Yes	594	89%	2.18	1.33–3.59	
**Perceived self efficacy**						
	No	124	82%	1.00		0.035
	Yes	601	89%	1.73	1.03–2.91	

Table 4 shows the results of the multivariate analysis. Factors associated with taking one or more preventive measures were: 1) young age; 2) having been vaccinated against seasonal influenza; 3) having had seasonal influenza in the last five years; 4) a higher number of preventive measures regarded as effective; 5) low standards of education. Being young adults (i.e. aged 18 to 29) increased the adoption of precautionary action by a factor 8.

**Table 4:** Multivariate logistic regression analysis: predictors of taking one or more preventive measures among six listed precautions

		**OR**	**95%**	**CI**	**p-value**
**Age group**					
	18-29 years	7.90	1.01	16.58	0.04
	30-59 years	0.87	0.52	1.47	0.62
	60+	1.00			
**Vaccinated against seasonal influenza**					
	Yes	4.44	2.00	9.86	<0.001
	No	1.00			
**Infected with seasonal influenza in the past five years**					
	Yes	1.84	1.12	3.00	0.02
	No	1.00			
**Score for effectiveness of precautionary measures (scale 0–6)**					
		1.51	1.27	1.79	<0.001
		1.00			
**Educational level**					
	High school or more	0.57	0.33	1.00	0.05
	Not high school graduate	1.00			

## Discussion

Many respondents stated that they had taken precautions: 87% did so after the interviewer had detailed possible precautions one by one. When possible precautionary measures were listed by the interviewer, “precautions taken” scored 2.02 reflecting that precautionary behaviour, albeit frequent, focuses on a small number of preventive measures taken at a single time. Whatever the way of asking the question, the main precautions that the respondents stated they had taken (hand washing and avoiding crowded places) were in agreement with official recommendations [26]. In several surveys, hand washing was the main precaution that the respondents said they had taken [4,27]. This precaution was taken to the same extent on Reunion Island (59%) and in France (59.7%) but to a lesser degree in the Netherlands (36%). Avoiding gatherings (crowded places) was mentioned more often by the respondents on Reunion Island (34%) than in France (14.6%) [27] or the Netherlands (8%) [4].

The specific behaviour of young adults is an important result of our study. Young adults (18–29 years) appeared as being the most active in terms of prevention, in contrast to other studies which reported elderly people to be more active in applying preventive measures [5,28,29]. Young adults were 8 times more likely to undertake preventive measures than other age groups. At the same time, the proportion of the population reporting influenza like illness in our sample was higher among young people and decreased with age. This feature was in agreement with results of the virological survey conducted on the same population in the context of the CoPanFlu prospective study that revealed that young people had been far more frequently infected by influenza A (H1N1) than elderly respondents [16]. One interpretation might be that young adults could have reacted well once they realized that they were more exposed to influenza A (H1N1) than other age groups. The research into behavioural attitudes over an extended period during influenza epidemics shows that people are highly adaptive and that their attitudes evolve over time [4,5].

Moreover, the most educated respondents had likely adjusted their behaviour to the changing general belief about the real severity of the epidemic. Those people most informed about the projected lower severity of the epidemic, felt it less necessary to take effective precautions. Conversely, a low standard of education was found to be associated with precautionary behaviour in our study as in a similar study conducted in the Netherlands during a human avian influenza epidemic [5].

The vast majority of respondents said that they were well informed (78%) and showed that they were knowledgeable. Knowledge of modes of transmission scored a satisfactory 4.47 (on a scale of 0 to 6) especially since for each wrong answer one point was deducted from the total score. This was true for 40% of the respondents who mentioned the mosquito as a potential vector of transmission. The recent chikungunya epidemic in Reunion Island may explain this link between mosquito and influenza A (H1N1). Finally, a question remained as to the transmission of the infection from pigs to people. In fact the zoonotic transmission was exactly the opposite in Reunion Island and was that of a reverse zoonosis (ie: transmission of the virus from humans to animal) as large and prolonged contamination of swine herds in Reunion Island occurred as a consequence of the H1N1 pandemic [30].

In our study, multivariate analysis showed that vaccination against seasonal influenza appeared related to a more general preventive attitude, as has also been observed in other studies [31]. This suggests that seasonal vaccination applied before the start of the epidemic, as an initial step towards prevention, had promoted preventive behaviour during the epidemic. Similarly a previous study has shown that undertaking influenza vaccination in the past, greatly facilitates vaccination in subsequent years [32]. Vaccination against seasonal influenza was not part of the recommendations against influenza A (H1N1) and no specific vaccine against influenza A (H1N1) was available during the whole H1N1 outbreak in Reunion Island. Nevertheless, 37% of respondents, regardless of their age, believed in the efficacy of the vaccine against seasonal influenza and 14% stated that they had taken this precaution to protect them from influenza A (H1N1). This attitude fitted the logic of previous messages: a vaccine that includes strains that circulated in previous years is also supposed to provide a protection (even partial) against the newly introduced strain. This was what transpired during the epidemic [16]. Vaccination against seasonal influenza was more widely adopted in main France than in the overseas department of Reunion Island (25.6% versus 14%). Of note, however, is the fact that in France, where the specific vaccine against influenza A (H1N1) was available, the use of the new vaccine was limited with a coverage of only 27.4% [25].

The number of preventive measures regarded as effective scored 3.04 (on a scale of 0 to 6), confirming good knowledge of preventive measures and confidence in the precautions suggested in the local prevention campaigns. The score for the effectiveness of preventive measures remained a predictor of precautionary behaviour in the model. The optimism through belief in a large number of precautions regarded as effective was also shown in another study conducted in the United Kingdom [2].

Perceived severity and vulnerability with regard to influenza A (H1N1) were regarded as moderate with average scores of 6.05 and 5.26 respectively (on a scale of 0 to 10). As our survey was conducted after passage of the epidemic wave, the perception of risk and severity of influenza A (H1N1) might have been minimized. A previous survey conducted in three phases over the course of an epidemic showed that perceived severity decreased at the end of the epidemic [4]. However, these perceptions remain higher on Reunion Island than in France where perceived severity and vulnerability were on average 3.64 and 2.95 respectively [27]. Finally, the severity of influenza A (H1N1), although rated as moderate, may have been slightly overestimated by our sample owing to the fact that it contained a large proportion of women whose perceived severity and perceived vulnerability were observed to be higher. This finding is shared by other studies [5,33]. Influenza A (H1N1) was regarded as more serious than seasonal influenza by most of the respondents (58%) but did not cause major worries compared to other concerns. Health concerns focused on chronic diseases; cancer was the main concern of the respondents, as had been observed in a previous study in France [33]. Moreover, influenza A was presented in the media as a serious epidemic, meaning that a parallel could have been drawn with the unquestionably more severe vector born Chikungunya epidemic. This potential danger was confirmed by concern over a future chikungunya epidemic that scored 7.1 (on a scale of 0 to 10). The perceived risk of influenza A (H1N1) on Reunion Island must be interpreted in the context of the previous chikungunya epidemic of 2006. Although this epidemic occurred three years before, it rapidly spread on a large scale, was severely symptomatic in a large fraction of the population (muscular and articular pain) and brought about chronic symptoms in a significant proportion of infected persons (266,000 cases, 246 persons hospitalized in intensive care, some 40 maternity-neonatal infections and a total of 243 deaths) [19]. Comparatively, the influenza A (H1N1) epidemic was regarded as far less severe both in terms of its scale and its health consequences.

Our study shares, with several other univariate analysis studies, three main factors influencing the adoption of precautionary behaviour: perceived severity, perceived vulnerability and perceived self-efficacy [2,4,5]. These results are in accordance with the Protection Motivation Theory (PMT) [24] and Health Belief Model (HBM) [25]. These factors are not always retained following a multivariate analysis since they are closely related to age, gender and standard of education, all factors that are confusing in a multivariate analysis. The socio-demographic profiles of the samples impact the model, revealing distinctive features specific to each target population target. Our sample could have played this role, highlighting the contrast in behaviour between elderly and young adult respondents. The research into behavioural attitudes over an extended period during influenza epidemics shows that such attitudes evolve over time and that people are highly adaptive [4,5]. The authors agree that such perceptions (severity, vulnerability, perceived self-efficacy) are underpinned by anxiety which is itself dependent on the messages conveyed by health authorities and which are in turn relayed by the media [2,4,5,34].

Our study has a number of limitations. Firstly, our sample was not representative of Reunion Island’s population due to the inclusion of a high proportion of elderly people and women. Hence whether conclusions could be generalized to the whole population of Reunion Island may be questionable. The over-represented elderly people and women weigh towards a selection bias. An explanation is that the interviewers preferred home-based respondents, thereby limiting refusals but making the choice of the household’s reference person more difficult. Analysis by sex and age group do not reveal significant statistical differences except a higher perception of severity by women. However, selection bias (women and elderly over-represented) has simply revealed, in the multivariate analysis, the contrasted attitude of young people. Apart from this result, elderly people did not appear to have a particular attitude. Elderly people could have overestimated severity of influenza A (H1N1) because influenza is known as an important cause of morbidity and mortality among elderly people [35]. However, our study did not reveal a higher perception of the risks among elderly respondents. The elevated tendency towards isolation among our target population does not seem related to the composition of our sample although elderly respondents may naturally be more inclined towards isolation owing to their generally reduced mobility. However, the young and elderly people in our sample declared that they taken this precaution to an equal extent (avoidance of mixing in groups: crowds, transport).

A second limitation is that the survey started at least 2.5 months after the outbreak and recall bias is a possible limitation. It seems however that this bias was mitigated. As already mentioned, there is a good correlation between infected cases of influenza A (H1N1) among respondents and their serological status [16]. These explanatory factors are consistent with a low recall bias.

A third limitation may arise from the introduction of desirability bias by the two-phase questioning technique used to assess the implementation of precautionary measures. However, compared to self-reported precautions, the second method has allowed collecting more complete data. Indeed, the response rate is always higher when memory is prompted by exhaustively recalling all the available precautions. On the other hand, the respondents very often mentioned first the active steps they had taken individually (hand washing, wearing of a mask, being vaccinated). The avoidance type precautions (not going to concerts, not traveling on public transport, not taking children to school) are retained more after a recall. It is possible that the reported precautionary behaviors of young adults could result from a social desirability bias as the participation of an interviewer can prompt responses reporting the implementation of prevention measures. Desirability bias is unlikely once the reality of the epidemic could be clearly perceived at the end of the epidemic passage, especially if one considers the highly alarmist announcements released by health authorities and media at the start of the epidemic.

Fourthly, the proportion of the population taking precautionary behaviour is high (87%) and interpretation of differences by predictor is challenging. The lack of power of the statistical test is balanced by a high size of the sample and a high participation rate observed (95%).

Our study has also a number of strengths. Firstly, the high participation rate is unusual. For example, the participation rate, for a telephone survey, was 46% on a representative sample of the population in France (excluding French overseas territories) [25]. In the Netherlands during an Internet-based survey (online questionnaire), the participation rate improved during the epidemic: respectively 59% at the start of the epidemic, 63%, half way through and 79% at the end [4]. In Reunion, the survey on perceived risks and preventive attitudes was conducted on average 2.5 months (SD 1.5) after the epidemic ended. This fact may explain the high participation rate observed. A further explanation may be that the respondents in our sample had been contacted regularly during the A (H1N1) epidemic as part of the CoPanFlu-RUN protocol (virological aspects) [16,21,22].

A second strength of this study is that the proportion of population (20–59 years) reporting influenza like illness (42%) in our sample was comparable to the seroconversion rates observed (39.4%) in the prospective serosurvey [16].

Thirdly, the percentages obtained from dichotomous questions (yes/no) from our questionnaire appear consistent with the scores obtained by combining several separate responses. This observation is reassuring with regards to the relevance of our sample analyses.

## Conclusion

In our study, the degree of severity of the epidemic was well estimated by the population despite initial alarmist messages. Precautions that were undertaken appeared in line with the degree of severity of the epidemic and with public health recommendations. Young adults, the age group the most exposed to influenza A (H1N1), reacted well. The most educated respondents seemed to have adjusted their behaviours to the epidemic severity. Moreover, it is reasonable to think that the chikungunya epidemic of 2006 brought an experience to the entire population of Reunion Island, as well as health professionals and health authorities, to face emerging epidemic diseases like the H1N1 epidemic in 2009.

Our study provides relevant and useful information to future preventive campaigns. It is important to fine tune national messages on prevention to adapt local situations. Our study shed light on the confusions with regard to the role of mosquitoes in transmission of influenza A (H1N1) virus. It was important to identify and remove this confusion in order to encourage the population to take more appropriate and targeted precautions. While it is necessary to combat unsound and unsubstantiated beliefs, our findings have also revealed the importance of informing the population about effective measures. This research illustrates the complexity of peoples’ understandings and responses to health messages diffused on the H1N1 pandemic. Further qualitative studies are needed to adapt messages to social and cultural realities of diverse populations and to prevent misconceptions.

The belief in a large number of precautions regarded as effective was a key factor in the precautions that were taken. Rallying people around effective measures and shifting of attitudes entails a continuous flow of accurate, truthful and targeted communication by health authorities before and during the epidemic. The inducement of preventive attitudes before epidemics seemed to promote and encourage the precautionary action taken during the epidemic.

## Endnote

^a^ Data not shown.

## Competing interests

The authors declare that they have no competing interests.

## Authors’ contributions

FT, MC and KD conceived and designed the study, analyzed the data and wrote the manuscript. FF, CD played a key in collecting the data. AF played a key role in entering the data. FF, FC reviewed the manuscript and contributed to subsequent drafts. FF acted as guarantor. All the authors proofread and approved the final manuscript.

## Pre-publication history

The pre-publication history for this paper can be accessed here:

http://www.biomedcentral.com/1471-2334/13/34/prepub
